# Vest-Pocket Anatomist

**Published:** 1892-02

**Authors:** 


### Book Reviews.

THE VEST-POCKET ANATOMIST.—(Founded upon "Gray."—By C. Henri Leonard, A. M., M. D., Professor of Medical and Surgical Diseases of Women, and Clinical Gynecology, Detroit College of Medicine, etc., etc.; Fourteenth Revised Edition, Containing Dissection Hints and Visceral Anatomy.

This is a small book of 296 pages, thoroughly illustrated with plain and colored plates of human anatomy, with explanations of proceedures necessary in regular dissections. As a book for ready reference it will prove valuable, both to the student and the busy practitioner. The plates are clear and distinct, and second only to Gray for the purposes they are intended. R. W. W.

